# CAR-T cell therapy for pancreatic cancer: Translating emerging targets and dual-targeting strategies from solid tumors

**DOI:** 10.3389/fimmu.2026.1764452

**Published:** 2026-04-10

**Authors:** Shijun Shen, Zhengcai Ruan, Beier Jiang, Wenjing Qiu, Feng Zhang, Runzhe Shu

**Affiliations:** 1 Department of Hepatopancreatobiliary and Minimally Invasive Surgery, The People’s Hospital of Lincang, The Eighth Affiliated Hospital of Dali University, Lincang, China; 2 Department of Science and Education, The People’s Hospital of Lincang, The Eighth Affiliated Hospital of Dali University, Lincang, China; 3 Zhejiang Provincial Engineering Research Center for Endoscopic Instruments and Technology Development, Quzhou Affiliated Hospital of Wenzhou Medical University, Quzhou People’s Hospital, Quzhou, China; 4 Australian Regenerative Medicine Institute, Monash University, Clayton, VIC, Australia

**Keywords:** cancer targets, CAR-T, dual CAR-T cells, immunotherapy, pancreatic cancer, solid tumor, tumor microenvironment

## Abstract

Pancreatic ductal adenocarcinoma (PDAC) is regarded as one of the most lethal malignancies, characterized by a poor prognosis and significant resistance to conventional treatments. Although Chimeric Antigen Receptor (CAR)-T cell therapy has been considered to be a revolutionary treatment for B-cell malignancies, its efficacy against solid tumors, including PDAC, has been limited. Nevertheless, after numerous tests pre-clinically and clinically, the acceptance of the first New Drug Application (NDA) for a CAR-T therapy in a solid tumor has sparked considerable hope and interest, which could further accelerate the field. Unlocking the full potential of CAR-T in PDAC requires overcoming significant hurdles, primarily the lack of ideal tumor-specific antigens and the profoundly immunosuppressive tumor microenvironment (TME). Given the shared expression of tumor-associated antigens (TAAs) across diverse solid tumors, this review analyzes promising solid tumor targets to identify candidates with high translational viability for PDAC. We summarize the latest clinical progress of CAR-T cell therapy, highlight emerging therapeutic targets, and explore innovative engineering strategies for developing potent, multi-targeted CAR constructs that are advancing toward future clinical application.

## 1 Introduction

According to the latest global cancer statistics from 2022, pancreatic cancer is the seventh leading cause of cancer death worldwide, with one of the lowest five-year survival rates among common cancers (1). Pancreatic ductal adenocarcinoma (PDAC) accounts for the vast majority of these cases and remains one of the world’s most lethal malignancies. Most patients are diagnosed at advanced stages, and projections indicate its public health burden will continue to grow (2). This grim prognosis is not merely due to late diagnosis but is deeply rooted in the aggressive biology of the disease. PDAC is characterized by a dense extracellular matrix (ECM), known as desmoplasia, which creates a physical barrier that limits drug delivery and promotes a profoundly immunosuppressive tumor microenvironment (TME). Moreover, the complex molecular mechanisms underlying PDAC tumorigenesis, including multiple genetic mutations and deregulated signaling pathways, contribute to its highly heterogeneous nature, making it hard to treat with any single targeted therapy (3, 4). These factors make the disease highly intractable and underscore the urgent need for innovative therapeutic strategies.

## 2 PDAC: pathogenesis and therapeutic strategies

**Figure 1:**
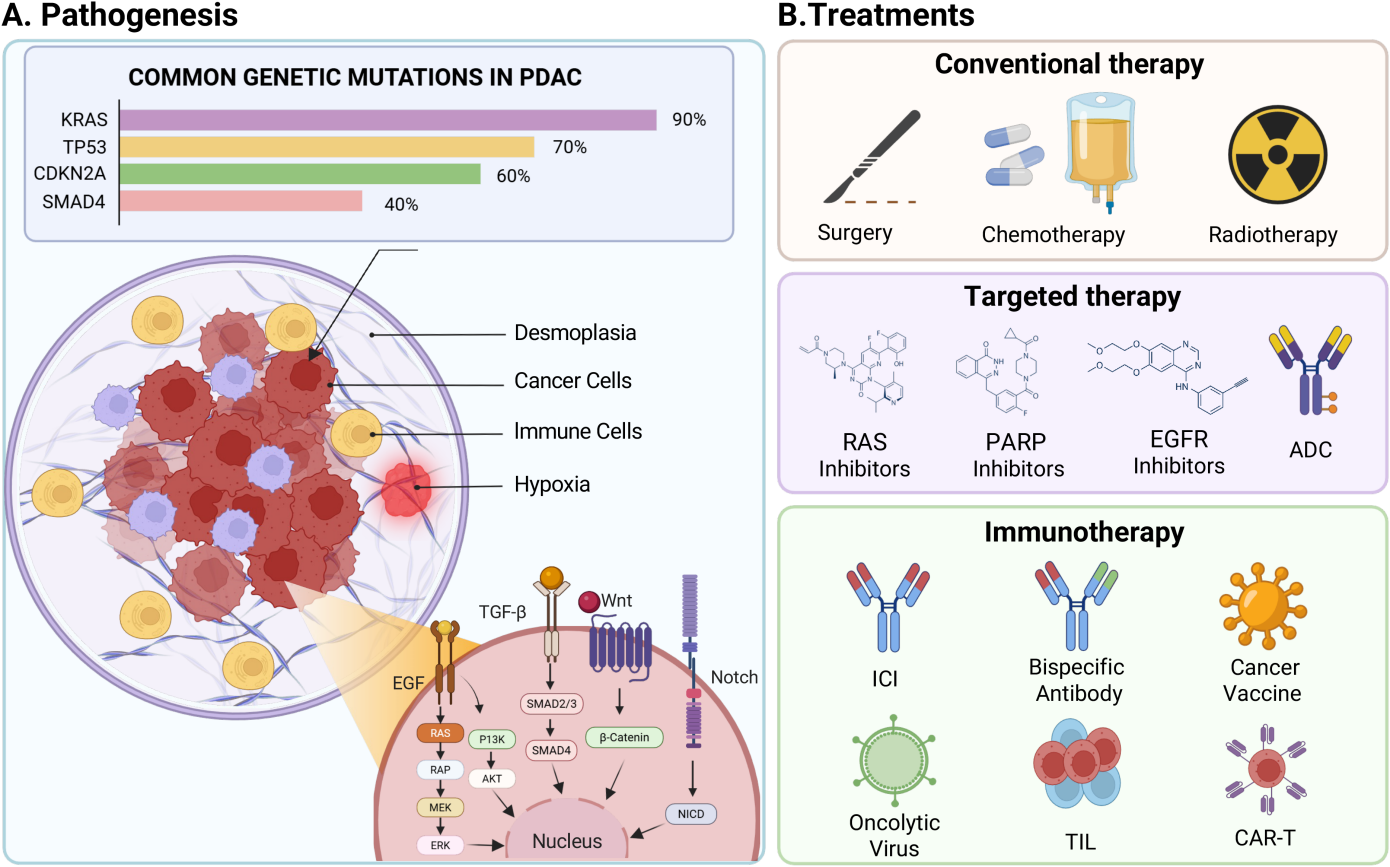
Pathogenesis and evolving therapeutic strategies for PDAC. **(A)** PDAC is featured as a desmoplastic, suppressive and hypoxic tumor microenvironment (TME). The development of PDAC is driven by the accumulation of key genetic mutations such as KRAS, TP53, CDKN2A, and SMAD4 and dysregulation of major signaling pathways like EGF, TGF-β, Wnt/β-Catenin and Notch. **(B)** Current and investigational treatments can be categorized into three tiers. Conventional therapies include the standard of care: surgery, multi-agent chemotherapy regimens (e.g., FOLFIRINOX, Gemcitabine/nab-paclitaxel) and radiotherapy. Targeted therapies utilize small molecules targeting specific pathways, such as EGFR, RAS, PARP, and VEGFR; or utilize antibody drug conjugate (ADC) through surface protein recognition. Immunotherapies represent novel modalities, including immune checkpoint inhibitors (ICIs), bispecific antibodies (BsAbs), cancer vaccines, oncolytic virus, tumor-infiltrating lymphocytes (TILs), and chimeric antigen receptor T cell (CAR-T) therapy.

PDAC is characterized by a stepwise progression of genetic alterations and a uniquely aggressive tumor microenvironment (TME) (**Figure 1A**). The genetic landscape of PDAC can be categorized into primary driver mutations and other actionable genetic alterations. The disease is dominated by KRAS mutations, which are present in approximately 90% of cases and are also considered as the primary driver for tumor initiation and maintenance by activating downstream signaling cascades such as the RAF-MEK-ERK and PI3K-AKT-mTOR pathways (5, 6). PDAC development is also driven by concurrent mutations in key tumor suppressor genes, including TP53 (70%), CDKN2A (60%), and SMAD4 (40%), which disrupt cell cycle regulation and promote proliferation (7).

In addition to the driver mutations, a distinct subset of PDAC tumors harbors actionable mutations in DNA damage repair (DDR) genes, most notably BRCA1 and BRCA2. The prevalence of somatic and germline mutations in DDR pathways in metastatic PDAC is reported to be approximately 15–25% (8, 9). Rather than initiating tumorigenesis in the same manner as KRAS, these abnormalities in DDR pathways are closely associated with malignant progression, and upregulation of these pathways is linked with treatment resistance. However, these alterations also establish unique biological vulnerabilities, rendering this subset of tumors sensitive to targeted therapies such as PARP inhibitors (8, 9). This complex genetic landscape, combined with dysregulation of core signaling pathways, such as epidermal growth factor receptor (EGFR), Wnt/β-catenin, and Notch, constitute a complex mechanistic network underlying PDAC tumor niche, and leads to the formation of a fast-growing, desmoplastic stroma with a hypoxic and immunosuppressive TME. It not only acts as a physical barrier impairing drug delivery but also fosters a heterogeneous tumor profile contributing to intrinsic therapeutic resistance and cancer recurrence (4, 7).

Like most solid tumors, conventional treatments for PDAC include surgery and chemotherapy (**Figure 1B**). Upfront surgery followed by adjuvant treatment is still one of the potential cures for early-stage patients. However, less than 20% of patients are diagnosed with resectable tumors, and even after surgery, recurrence rates remain high (10). In most settings, PDAC patients are at advanced stage with metastatic disease (11). The current standard-of-care for advanced PDAC relies on chemotherapy regimens like FOLFIRINOX, gemcitabine-based combinations or NALIRIFOX, which offer only modest survival benefits, typically under one year for metastatic disease (12–14). Targeted therapies, such as KRAS G12C inhibitors (e.g., Sotorasib), EGFR inhibitors (e.g., Erlotinib), PARP inhibitors (e.g., Olaparib for BRCA-mutated cases), are applicable to only a small subset of patients (15). Therefore, emerging therapies for PDAC are rapidly evolving to enhance precision and avoid resistance from other cancer indications. Antibody-drug conjugates (ADCs) and bispecific antibodies (BsAbs), both have new drugs approved for other cancer indications (16–18). Oncolytic viruses (OVs) and mRNA neoantigen vaccines are showing promise in stimulating durable anti-tumor responses (19, 20). Critically, ICIs that have revolutionized the treatment of other cancers have largely failed in PDAC. This is because pancreatic cancer is considered an “immunologically cold” tumor (21). PDAC is characterized by a low tumor mutational burden (TMB), which results in a scarcity of neoantigens required for endogenous T cells to recognize and attack cancer cells (22). Consequently, ICIs, which work by “releasing the brakes” on a pre-existing anti-tumor immune response, are ineffective in most PDAC patients because there is often no significant immune response to unleash (23, 24).

Failure of ICIs, that rely on activating endogenous immunity creates a clear and urgent rationale for a different approach: adoptive cell therapy (ACT). This strategy involves collecting a patient’s T cells, expanding or engineering them *ex vivo* and re-infusing them as a “living drug”. Several ACTs have now been approved for solid tumors, validating this approach (25, 26). These include Tumor-Infiltrating Lymphocyte (TIL) therapy, where naturally tumor-reactive T cells are isolated from a patient’s tumor, expanded to large numbers, and re-infused, which has led to an approval for advanced melanoma by U.S. Food and Drug Administration (FDA) (27). Another successful strategy is T-cell receptor (TCR)-T therapy, where T cells are engineered to express a specific TCR that recognizes intracellular tumor antigens presented on the cell surface by major histocompatibility complex (MHC) molecules, and this approach has been validated with the FDA approval of a MAGE-A4 directed TCR-T product for synovial sarcoma (28). Within this landscape, Chimeric Antigen Receptor (CAR)-T cell therapy offers a distinct and powerful tool.

## 3 CAR-T cell therapy

### 3.1 Basic designs and engineering strategies

The core principle of CAR-T therapy is to engineer T cells with synthetic receptors (CARs) that recognize specific tumor-associated antigens (TAAs) on the cancer cell surface, independent of MHC presentation (29). A standard CAR construct consists of an extracellular antigen binding domain (e.g., Single-chain variable fragment (scFv) or Variable heavy domain of heavy chain (VHH)), hinge and transmembrane domains (e.g., CD28, CD8a, CD4), as well as the intracellular T cell signaling domains. The most widely used design is the 2^nd^ generation CAR, which incorporates a co-stimulatory domain (e.g., CD28, 4-1BB) alongside the CD3 T cell activation domain.

**Figure 2:**
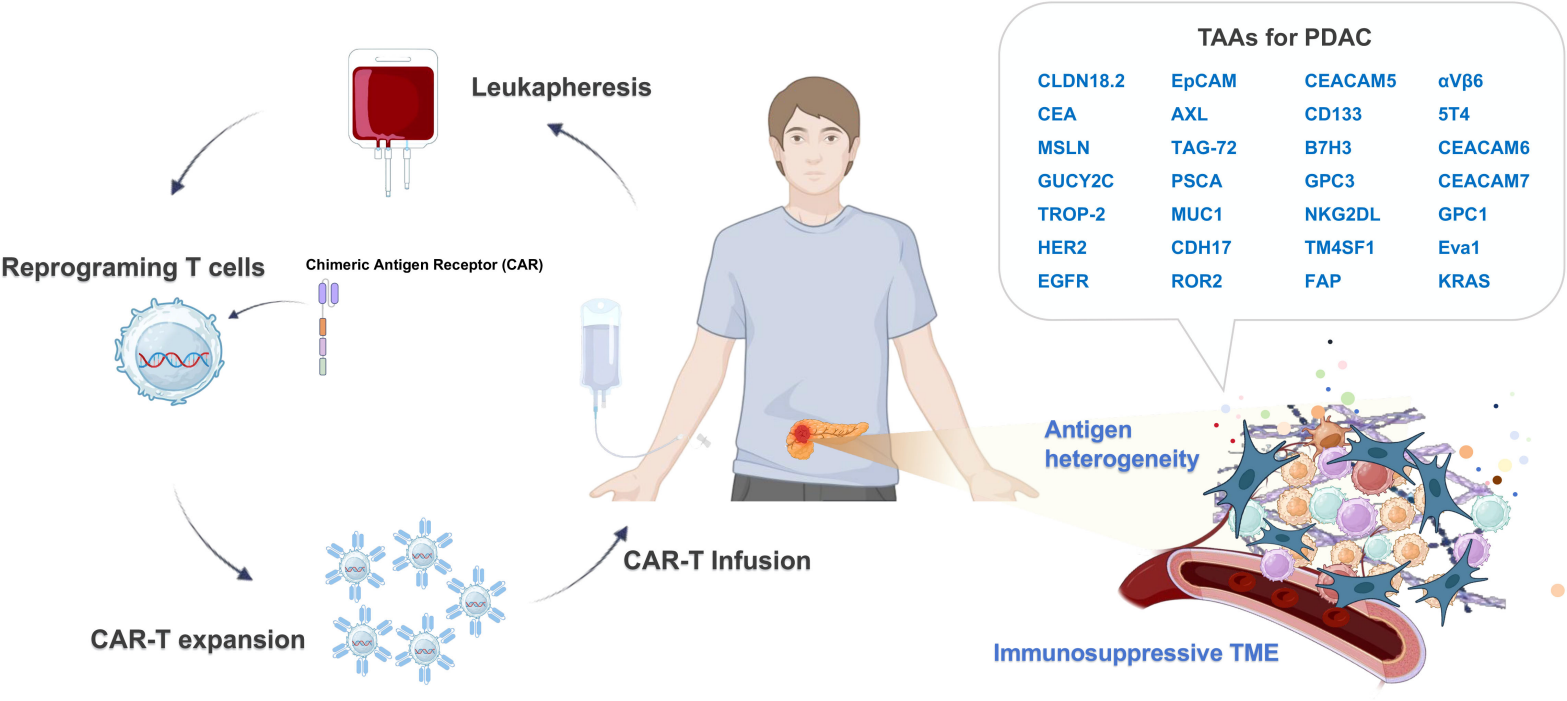
Overview of CAR T-cell therapy and potential tumor-associated antigens (TAAs) for Pancreatic Ductal Adenocarcinoma (PDAC). PDAC features a complex stromal microenvironment characterized by a dense extracellular matrix (ECM) and an immunosuppressive tumor microenvironment (TME), composed of cancer-associated fibroblasts (CAFs) and immunosuppressive immune cells such as tumor-associated macrophages (TAMs), regulatory T cells (Tregs), and myeloid-derived suppressor cells (MDSCs). Autologous T cells are collected from the patient’s peripheral blood via leukapheresis, followed by activation and introduction of the chimeric antigen receptor (CAR) gene, typically using viral vectors or non-viral electroporation. After *in vitro* expansion and quality assessment, the CAR-T cells are administered to the patient through systemic or regional delivery. The infused CAR-T cells selectively bind to TAAs expressed on the surface of cancer cells and subsequently eradicate the cancer cells.

The CAR-T therapy process involves collecting autologous T lymphocytes from the patient, which are then activated, genetically engineered to express the CAR, and expanded in the laboratory before being reinfused back into the patient, locally or systemically (30, 31). While CAR-T therapy has achieved remarkable success in B cell malignancies, its efficacy in solid tumors like PDAC is severely compromised. To address this, multiple engineering strategies are being deployed to enhance T-cell fitness, persistence and potency (32). For example, armored CAR-T cells are engineered to secrete pro-inflammatory cytokines like IL-12 or IL-18 (33, 34). Additionally, gene editing technology like CRISPR-Cas9 can be utilized to knockout inhibitory genes like PD-1 (35). As CAR-T approach is able to transfer a large, targeted, and highly potent effector cell population into the patient, it represents a critical strategy designed to overcome the low immunogenicity and hostile TME of PDAC (**Figure 2**) (36).

### 3.2 T cell subpopulations and functional heterogeneity

CAR-T products comprise heterogeneous subpopulations of T cells, and variations in manufacturing protocols, such as the expansion time and cytokine supplementation (e.g., IL-2 vs. IL-7/IL-15), can alter the final composition of the CAR-T cell product. However, many published CAR-T clinical trials administered bulk T cells without thoroughly defining the final cellular composition. Consequently, patients received an unknown ratio of CD4+ to CD8+ cells, as well as highly variable mixtures of naïve, effector, and memory T cells. It is now widely recognized that the specific subtypes of T cells within the CAR-T product dictate the *in vivo* kinetics, persistence, and overall efficacy as well as the safety profile of the treatment, particularly in the challenging context of solid tumors (37).

In the natural immune system, CD8+ T cells act as cytotoxic T lymphocytes (CTLs) that directly kill malignant cells, while CD4+ T cells primarily function as “helpers” that orchestrate the immune response through cytokine secretion. The CAR construct could bypass traditional MHC restriction, equipping CD4+ CAR-T cells with direct cytotoxic capabilities (37). CD8+ CAR-T cells are rapid and highly efficient killers but are prone to aggressive hyper-proliferation and functional exhaustion. Data from TIL therapy suggest that a higher number of CD8+ T cells correlates with better clinical response in solid tumor. TIL responders are infused with predominantly tumor-resident CD8+ T cells, which preferentially re-infiltrate tumors, though subsequent IL-2 administration is required to support persistence (38, 39). On the other hand, CD4+ CAR-T cells are slower killers but exhibit superior expansion, long-term persistence, and resistance to exhaustion. They secrete vital cytokines like IL-2 and IFN-γ that sustain CTL proliferation and recruit other infiltrating lymphocytes, such as endogenous natural killer (NK) cells, macrophages, and non-engineered host T cells, which is crucial for a comprehensive anti-tumor response. This secondary infiltration helps remodel the suppressive TME and can trigger “by-stander killing”, where the host’s own immune system begins to recognize and attack diverse secondary tumor antigens, further amplifying the therapeutic effect beyond the initial CAR-T target (40, 41).

Beyond CD4 and CD8 lineages, efficacy relies heavily on the T cell differentiation state. In CD19-directed CAR-T trials for hematological malignancies, manufacturing processes are being optimized to preserve early memory phenotypes by expanding cells with IL-7/IL-15 and utilizing 4-1BB costimulatory domains, this allows these cells to better infiltrate the bone marrow and lymph nodes, providing a continuous supply of new effector cells while resisting exhaustion. The stemness and memory of T cells could determine the *in vivo* expansion and clinical response in CD19 CAR-T products, and it can also reduce the manufacturing time and dose required to improve the safety for B-cell lymphoma patients (42, 43). Stem cell memory CAR-T cells have also exhibited better tumor control due to long-term persistence in pre-clinical solid tumor models (44), though stem cell or central memory T cells which preferentially traffic to secondary lymph nodes and bone marrow may need to be optimized for solid tumor infiltration via gene editing (45, 46). Alternatively, to maximize acute cytotoxicity, large doses of highly active, effector-like CAR-T cells can be utilized to rapidly debulk solid tumors. This strategy frequently employs CD28 costimulatory domains, which drive explosive, immediate effector function and rapid cytokine release (47, 48). Although these effector cells lack long-term persistence, this limitation can be mitigated through repeated infusions or cytokine armoring ensuring a continuous high response against the solid tumor (49).

### 3.3 Toxicity and safety management

While CAR-T cell therapy has demonstrated remarkable efficacy in blood cancers, it is associated with severe toxicities. The most prominent of these are Cytokine Release Syndrome (CRS) and Immune Effector Cell-Associated Neurotoxicity Syndrome (ICANS). While CRS is characterized by fever, hypotension, hypoxia and dyspnoea, ICANS can cause encephalopathy, aphasia, and seizures (50). Furthermore, the lymphodepleting conditioning chemotherapy (typically a regimen of fludarabine plus cyclophosphamide, FluCy) administered prior to CAR-T cell infusion introduces its own substantial toxicities which may cause prolonged cytopenias and heighten the patient’s risk for severe infections (51). Despite the high incidence and severity of these adverse events in blood cancers, they can now be highly controlled. Extensive clinical experience has led to standardized, highly effective management paradigms. The timely administration of tocilizumab and systemic corticosteroids has proven to rapidly mitigate both CRS and ICANS, effectively rescuing patients from life-threatening systemic inflammation without completely abrogating the therapeutic efficacy of the infused T cells (52).

As CAR-T therapy expands into the realm of solid tumors, clinical trials have shown that CRS and ICANS occur with lower incidence and severity compared to hematological malignancies, and these toxicities can be effectively controlled based on the guidelines established from the blood cancer experience (53). However, extra supportive care should be taken, as solid tumor patients are often older and have reduced bone marrow reserve due to years of prior cytotoxic therapy. Especially, incidence and severity of systemic inflammation will increase for patients with high tumor burden or high tumor antigen expression. Depending on the targeted antigen and the tumor’s anatomical origin, specialized toxicities can emerge. For instance, PDAC-directed CAR-T therapies may carry the unique risk of inducing severe, localized collateral damage, such as acute pancreatitis or severe gastrointestinal mucosal inflammation (54). In addition, patients with hematological cancers have a distinct immune microenvironment compared to solid cancer patients; as a result, the lymphodepletion regimes used for blood cancers should be modified based on the tumor types and patients’ conditions. The dose of FluCy can be reduced to avoid bone marrow suppression, and additional agents, such as nab-paclitaxel, can be considered to suppress the TME before CAR-T infusion (53). Ultimately, the most crucial and dangerous challenge in solid tumor CAR-T therapy is on-target/off-tumor (OTOT) toxicity. Because most solid tumor targets are also expressed at low levels on healthy tissues, targeted immune activation can lead to catastrophic organ inflammation and even fatal tissue damage, which fundamentally limits the administered cell dose and severely narrows the therapeutic window (55, 56).

### 3.4 Target selection

The success of CAR-T therapy for PDAC is fundamentally dependent on the identification and validation of superior tumor-associated antigens (TAAs). The ideal target should be highly and uniformly expressed across all cancer cells but completely absent from essential normal tissues. However, such perfect targets, known as tumor-specific antigens (TSAs), are exceedingly rare. Therefore, the continuous discovery of new targets is crucial for several reasons. Firstly, it expands the pool of patients who may be eligible for CAR-T therapy and other antibody-based therapies. Secondly, multiple antigen-dependent mechanisms such as antigen heterogeneity, antigen downregulation, shedding, and epitope loss, can result in tumor recurrence; a broader portfolio of validated targets is the foundation for developing multi-targeting CAR-T cells to overcome these challenges and prevent tumor escape (57). Finally, identifying targets with better tumor-to-normal tissue expression ratios is the most direct way to improve the safety profile and mitigate the risk of severe OTOT toxicity (58, 59). Consequently, the ongoing search for novel targets represents a critical path forward to unlock the full therapeutic potential of CAR-T cells in PDAC.

## 4 Clinical-stage CAR-T targets for PDAC

One of the major reasons for the success of CD19 CAR-T was attributed to the tolerability of B-cell depletion along with the malignant cells. This is not the case for other tumors; the majority of CAR-T cell therapy targets for solid tumors are TAAs that are also expressed at low levels on normal tissues (60). Because most of the PDAC TAAs are shared across various solid tumors, given the aggressive nature and rapid progression of PDAC, initial clinical trials often enroll patients with other less aggressive malignancies to establish baseline safety, optimal dosing and preliminary efficacy before progressing to PDAC cohorts. Consequently, robust clinical data exclusive to PDAC remains limited. To bridge this translational gap, **Table 1** summarizes key clinical-stage targets applicable to PDAC. In the following section, we highlight and discuss several of these targets that have already demonstrated encouraging clinical efficacy and manageable toxicity profile in other solid tumors. By analyzing these broader clinical outcomes, we aim to provide a predictive blueprint for translating these highly promising candidates into effective, targeted interventions specifically for PDAC.

**Table 1:** Potential CAR-T Targets for PDAC at clinical stage.

Target	Applicable cancer	Highest phase	Representative clinical trials	Clinical outcome	Reference
CLDN18.2	Gastric cancer, Gastroesophageal junction cancer, Pancreatic cancer, Colorectal cancer, Signet ring cell adenocarcinoma	Phase 2/3	NCT03874897, NCT04581473, NCT07103668	Advanced GC: ORR 38.8%, DCR 91.8%; mPDAC: ORR 16.7%, DCR 70.8%.	(61–65)
CEA (CD66)	Colorectal cancer, Pancreatic cancer, Hepatocellular carcinoma, Adenocarcinoma	Phase 2/3	NCT06006390, NCT05396300, NCT02850536, NCT04037241	LM: Phase 1b: i.a CAR-T delivery showed mOS of 8 months with manageable safety. A Phase 2/3 trial was proposed but withdrawn; Solid tumors: Recent hypoxia-responsive CAR-T Phase I trial show DCR of 82.4% in i.p group and 68.0% in i.v group.	(66–70)
Mesothelin (MSLN)	Pancreatic cancer, Ovarian cancer, Breast cancer, Lung cancer, Fallopian Tube Cancer, Mesothelioma	Phase 1	NCT01897415, NCT02159716, NCT02414269, NCT06249256	Solid tumors: Limited efficacy, stable disease as best outcome; MPM: PD-1 inhibitor combination therapy extends OS.	(71–75)
GUCY2C (GCC)	Metastatic pancreatic cancer, Advanced gastrointestinal Malignancies	Phase 1	NCT04652219, ChiCTR2100044831, NCT05287165, ChiCTR2000040645, NCT05319314	mCRC: 80% ORR at dose level 2 group.	(76–79)
TROP-2	Breast cancer, Pancreatic cancer, ovarian cancer, various solid tumors	Phase 1	NCT06082557	Advanced solid tumors: No clinical outcome reported.	(80)
HER2	Breast cancer, Lung cancer, Pancreatic cancer, HER2 positive solid tumors	Phase 1	NCT01935843, NCT06101082, NCT04995003, NCT00902044	PDAC: 1 PR, 5 SD in 11 patients; Sarcoma: 3 CR, 4 SD in 14 patients, and PD-1 inhibitor combination clinical trial is ongoing.	(81, 82)
EGFR	Non-small cell lung cancer, Pancreatic cancer; various solid tumors	Phase 1	NCT01869166, NCT06682793	Advanced BTC: 1 CR, 10 SD in 17 patients. Solid tumors: Logic-gated CAR-T Phase 1 trial was proposed.	(83–85)
EpCAM	Gastric cancer, Pancreatic cancer, Colorectal cancer, Digestive system cancers	Phase 1	NCT05028933, NCT02915445	Advanced GC: ORR 30%, DCR 70%.	(86)
TAG-72	Pancreatic cancer, Ovarian cancer, Gastric cancer, Colorectal cancer, Adenocarcinomas	Phase 1	NCT05225363, NCT06846424	CRC: 1st-gen CAR-T was safe but showed limited efficacy; OV: 2^nd^ gen CAR-T clinical trial via i.p delivery is ongoing.	(87, 88)
PSCA	Gastric cancer, Pancreatic cancer; Prostate cancer, Bladder cancer	Phase 1	NCT02744287, NCT03873805	mPDAC: DCR 60%, mCRPC: DCR 100%; Two treatment-related deaths, trial terminated due to DLT.	(89, 90)
IL13Rα2	Glioblastoma, Neuroendocrine tumors, Pancreatic neuroendocrine neoplasms, Pancreatic cancer	Phase 1	NCT02208362, NCT04119024	GBM: i.c delivery was well tolerated, 62% patients showed tumor regression.	(91, 92)
MUC1	Gastric cancer, Pancreatic cancer, Colorectal cancer, Breast cancer, Lung cancer	Phase 1	NCT05812326, NCT05239143, NCT04025216,	Solid tumors: Best outcome is SD, no SAE observed.	(93, 94)
CDH17	Biliary tract cancer, Colorectal cancer, Gastric cancer, Pancreatic cancer	Phase 1	NCT06055439, NCT06501183, NCT06937567	Solid tumors: No clinical outcome reported.	(95, 96)
ROR2	Bladder cancer, Gastric cancer, Pancreatic cancer, Renal cell carcinoma, Soft tissue sarcoma	Phase 1	NCT03960060, NCT03393936	Solid tumors: No clinical outcome reported. Trials terminated due to adjustment of study strategy.	(97)
CEACAM5	Breast cancer, Colorectal cancer, Gastric cancer, Lung cancer, Ovarian cancer, Pancreatic cancer	Phase 1	NCT01212887	Solid tumors: Poor persistence and respiratory toxicity. Trial terminated due to safety concerns and lack of efficacy.	(98)
CD133	Acute Myeloid Leukemia, Brain cancer, Breast cancer, Cholangiocarcinoma, Colorectal cancer, Hepatocellular carcinoma, Ovarian cancer, Pancreatic cancer	Phase 1/2	NCT02541370	HCC: 1 PR, 14 SD in 21 patients.	(99, 100)
B7H3	Breast cancer, Fallopian tube cancer, Ovarian cancer, Pancreatic cancer, Breast cancer	Phase 1	NCT06158139, NCT06515626, NCT05474378	PDAC: No clinical outcome reported. GBM: Preliminary mOS of 14.6 months and frequent TIAN after i.c delivery.	(101, 102)
GPC3	Hepatocellular carcinoma, Pancreatic cancer	Phase 1	NCT05779917, NCT06196294, NCT05155189 NCT03198546, NCT02395250	PDAC: No clinical outcome reported. HCC: Best clinical outcome: DCR 90.9%, ORR 50%.	(103–105)
TM4SF1	Colorectal cancer, Esophagus cancer; Gastric cancer, Lung cancer, Mesothelioma, Neoplasms, Ovarian cancer, Pancreatic cancer	Phase 1	NCT05673434, NCT04151186	Solid tumors: No clinical outcome reported. Trials under unknown status.	(106)
FAP	Malignant pleural mesothelioma, Breast cancer, Head and neck cancer, Lung cancer, Ovarian cancer, Pancreatic cancer	Phase 1	NCT01722149	MPM: Locally delivery of 1x 10^6^ CAR-T cells is well tolerated.	(107)

ORR, overall response rate; DCR, disease control rate; OS, overall survival; CR, complete response; PR, partial response; SD, stable disease; DLT, dose limiting toxicity; i.p, intraperitoneal; i.v, intravenous; i.c, intracranial; i.t, intratumoral; i.a intraarterial; GC, gastric cancer; BTC, Biliary tract cancers; MPM, malignant pleural mesothelioma; mPDAC, metastatic pancreatic ductal adenocarcinoma; mCRC, metastatic colorectal cancer; CRPC, metastatic castration-resistant prostate cancer; HCC, hepatocellular carcinoma; GBM, glioblastoma; TIAN, tumor inflammation-associated neurotoxicity; LM, liver metastases.

### 4.1 Claudin18.2

Claudin18.2 is an extremely popular industry target, and multiple antibody drugs are being developed for solid cancer treatment. Claudin 18 isoform 2 is a member of the claudin family and expressed in the cell membrane as a tight junction structure, and it is highly expressed in gastric, pancreatic, esophageal cancer and other adenocarcinomas (108). In the open-label Phase 1/1b clinical study (NCT03874897) of 98 patients with gastrointestinal malignancies, treatment with CLDN18.2 CAR-T achieved an overall response rate (ORR) of 38.8%, disease control rate (DCR) of 91.8%, median progression-free survival (mPFS) of 4.4 months, and median overall survival (mOS) of 8.8 months (61, 62). The recent pivotal Phase 2 trial (NCT04581473) for advanced gastric or gastroesophageal junction cancer, CLDN18.2 CAR-T cells administered up to three times at a dose of 2.5 × 10^8^ cells demonstrated superior efficacy compared to standard therapy. The treatment significantly improved progression-free survival (3.25 vs. 1.77 months) with a manageable safety profile. The objective response rate was 35%, versus 4% in the control group. Patients receiving CAR-T therapy also had a median overall survival advantage of 2.4 months and a 31% reduced risk of death (63). A pooled analysis of data from these trials also highlighted efficacy in metastatic pancreatic cancer, with an ORR of 16.7%, DCR of 70.8%, mPFS of 3.3 months, and mOS of 10.0 months (64). Notably, complete remission of target lesions was reported in select metastatic pancreatic cancer patients with high CLDN18.2 expression following CAR-T treatment (56, 109, 110). The CLDN18.2 CAR-T product (satri-cel) was filed for gastric cancer approval with National Medical Products Administration (NMPA) in China and could shortly become the world’s first CAR-T therapy to be approved for a solid tumor indication. Additionally, a distinct VHH-based CLDN18.2 CAR-T product exhibited a favorable safety profile and encouraging efficacy in the Phase1/2a study, leading to the initiation of a Phase 3 randomized controlled trial (NCT07103668) (65). Given the expression profile of CLDN18.2 in pancreatic cancer and comparable Phase I outcomes to gastric cancer, CLDN18.2 CAR-T is also poised to become the next approved CAR-T therapy for pancreatic cancer.

### 4.2 Carcinoembryonic antigen

CEA (CD66) is a diagnostic marker elevated in 30% to 60% of PDAC patients and a significant prognostic indicator (111). In normal tissues, its expression is largely restricted to the apical surface of gastrointestinal epithelial cells, making it relatively inaccessible to systemic immune cells. Early clinical trials of CEA-targeted CAR-T cells delivered via hepatic artery infusion for liver metastases showed limited efficacy, with most patients experiencing progressive disease, leading to the termination of a subsequent Phase 2/3 trial (66, 67). To overcome the limitations of systemic toxicity and poor persistence, a novel hypoxia-responsive (HR) PC13 CAR construct was engineered, which allows high CAR expression in hypoxic TME while low CAR expression in circulation and healthy tissues (112). Recent clinical studies evaluating these novel HR-CEA CAR-T cells have demonstrated highly encouraging results (70). In a phase 1 trial, intraperitoneal (i.p) delivery of PC13 CAR-T cells (up to 5 × 10^6^ CAR+ T cells/kg) achieved a disease control rate (DCR) of 82.4% and an objective response rate (ORR) of 23.5%. Notably, the clinical response was enhanced in a subgroup of patients with peritoneal metastases and high CEA expression (≥90%), reaching an ORR of 57.1% via i.p delivery. In another non-small cell lung cancer trial, HR-CEA CAR-T cells showed ORR of 47% and a DCR of 87% (68–70). These promising outcomes suggest that TME-regulated CAR constructs paired with locoregional delivery can effectively overcome the initial limitations of CEA-targeted therapy.

### 4.3 Mesothelin

Mesothelin (MSLN) is widely distributed and highly expressed in multiple solid tumors; approximately 80%-85% of pancreatic carcinomas express MSLN, while its expression in normal mesothelial cells is low. This profile has resulted in MSLN being one of the most studied targets for CAR T cells in PDAC patients (113). Extensive clinical investigations targeting MSLN, including for PDAC, have been performed, and MSLN CAR-T therapies were generally well tolerated with limited clinical efficacy (71, 74). MSLN expression might be upregulated in inflammatory lung conditions and led to severe pulmonary toxicity in the high-dose treatment cohort (73). The regional delivery of MSLN CAR-T combined with PD-1 inhibition is well tolerated and could further extend the median overall survival for malignant pleural mesothelioma (MPM) (72). A recent Phase 1 trial (NCT06249256) showed that MSLN CAR-T secreting PD-1 nanobody achieved ORR of 57% and DCR of 86% in 7 MPM patients (75). These studies indicated that regional administration of MSLN CAR-T cells could be a rational strategy to avoid neutralization, and the efficacy can be further enhanced by combined PD-1 blockade. In addition, upregulation of SOX4 and ID3 was linked to the T cell exhaustion in PDAC patient after MSLN CAR-T therapy, and this finding provided the rationale of knocking out SOX4 and ID3 to enhance CAR-T cell antitumor activity for PDAC (74).

### 4.4 Guanylate cyclase 2C

Guanylate Cyclase 2C (GUCY2C), also known as GCC, is a colorectal cancer (CRC) antigen that is also expressed in PDAC. A key characteristic that makes GUCY2C an appealing target is its inaccessibility in the apical membranes of polarized normal intestinal epithelial tissue, creating a unique therapeutic window for targeting metastatic lesions (114, 115). Building on the efficacy of GCC CAR-T cells in CRC mouse models, two clinical studies both showed acceptable safety profile as well as high ORR and DCR for advanced metastatic CRC (77, 78). Recent data for a sophisticated CAR-T product, GCC19 CAR-T, have been encouraging. GCC CAR-T cells were coupled with CD19 CAR expressing multiple cytokines. The CD19 CAR acted as a “helper” to boost the *in vivo* expansion of the entire CAR-T cell population upon encountering normal B cells. The Phase 1 trial using this product in patients with metastatic CRC demonstrated promising anti-tumor activity, with an ORR of 40% and a mOS of 22.8 months (76). Particularly in the US Phase 1 study, the ORR is 80% at the dose of 2 × 10^6^ cells/kg (4/5 with 3 PR and 1 CR) for refractory CRC patients (79). These results suggest that this advanced, multi-component “helper” CAR strategy can effectively boost the activity of solid tumor-targeting CAR-T cells and that GUCY2C remains a viable and promising target for PDAC.

### 4.5 Tumor-associated glycoprotein 72

Tumor-Associated Glycoprotein 72 (TAG-72), a high-molecular-weight mucin-like glycoprotein that is expressed on the surface of a wide variety of adenocarcinomas, including pancreatic, colorectal, gastric and ovarian cancers, while showing very limited expression in normal adult tissues (116). Multiple antibody clinical trials and a first-generation TAG-72 CAR-T clinical trial in patients with metastatic colorectal cancer established that this target was relatively safe (87, 116, 117). The second-generation TAG-72 CAR-T cells with different strategies were developed pre-clinically, such as regional delivery (88), IL-12 armored CAR (33), DGKα/ζ gene knock-out (118), TAG-72/CD47 dual CAR (119) and TAG-72 fusion protein T cells (120). These strong preclinical evidences have led to the initiation of Phase 1 clinical trials for patients with advanced ovarian cancer, but clinical outcomes have not yet been reported.

### 4.6 Prostate stem cell antigen

Despite its name, Prostate Stem Cell Antigen (PSCA) is also overexpressed in about 50% of metastatic PDAC besides 80% of prostate cancer. PSCA is associated with a more aggressive disease course and poorer survival with limited expression in normal epithelial cells, making it a suitable candidate for targeted therapy (121). Targeting PSCA with CAR-T cells offers a strategy to eliminate pancreatic cancer cells (122, 123). In Phase 1 dose escalation clinical trial (NCT02744287), a special designed PSCA CAR-T cells containing a 1^st^ generation CAR and inducible dual costimulatory MyD88/CD40 signal was investigated in metastatic PDAC and castration-resistant prostate cancer (mCRPC) patients. The DCR is 60% in mPDAC cohort and 100% in mCRPC cohort. However, two treatment-related deaths occurred in the highest-dose mCRPC cohort (89).

### 4.7 Cadherin-17

Cadherin-17 (CDH17) is a cell adhesion molecule that is highly expressed in gastrointestinal cancers, including pancreatic, gastric, and colorectal cancers. In normal tissues, CDH17 is predominantly localized to the lateral membrane and sequestered within the tight junctions of intestinal epithelial cells, making it largely inaccessible. This aberrant localization and high tumor expression makes CDH17 one of most appealing target for antibody-based therapeutics (124, 125). CDH17 CAR-T cells are able to eradicate CDH17-expressing neuroendocrine and gastric, pancreatic and colorectal tumors without OTOT to normal intestinal epithelial cells, which also express CDH17 (95, 126). A CAR-T product targeting CDH17 is currently in Phase 1/2 clinical trial to evaluate their safety and efficacy, and no clinical outcomes have been published (96).

### 4.8 Transmembrane-4-L-six-family-1

Transmembrane-4-L-six-family-1 (TM4SF1) is a cell-surface protein that is overexpressed in numerous solid tumors, including pancreatic cancer (127). It is believed to play a role in tumor cell proliferation, migration, and invasion (128). Its tumor-associated expression pattern makes it a viable candidate for CAR-T cell therapy (106). Phase 1 clinical trials (e.g., NCT05673434, NCT04151186) are currently underway to assess the safety and efficacy of TM4SF1-targeted CAR-T cells, but no clinical outcomes have been published.

### 4.9 Fibroblast activation protein

Cancer-associated fibroblasts (CAFs) are a major component of the dense desmoplastic stroma in PDAC. CAFs create physical barriers to immune infiltration and secrete immunosuppressive factors resulting in therapeutic resistance (129). Fibroblast Activation Protein (FAP) is predominantly expressed by CAFs within the tumor stroma, but also found on normal cells such as bone marrow stroma cells. Considering potential OTOT risk, low dose (1 × 10^6^) of FAP CAR-T cells were administered regionally in MPM patients, no severe toxicity was shown at this dose (107). As targeting FAP by CAR-T cells could remodel the hostile TME, targeting FAP was frequently considered in dual-targeting CAR-T strategies (130).

## 5 Pre-clinical stage CAR-T targets for PDAC

Due to the lack of tumor-specific surface antigens and the dose-limiting toxicities associated with shared TAAs, the scientific frontier in PDAC CAR-T research heavily involves identifying novel, next-generation targets to overcome existing therapeutic barriers. While the targets discussed in the previous section have entered clinical evaluation, the preclinical pipeline is critical for discovering antigens that offer a wider therapeutic window and deeper penetration into the immunosuppressive PDAC microenvironment. The following table and descriptions highlight key emerging targets currently in preclinical development (**Table 2**). Importantly, these preclinical evaluations, often leveraging *in vitro* and *in vivo* models across various solid tumor types, serve as the crucial foundation for developing dual-targeting CAR constructs designed specifically to overcome the challenges of pancreatic cancer.

**Table 2:** Potential CAR-T Targets for PDAC at Pre-clinical Stage.

Target	Disease indications	Protein function	Target notes	Prevalence in PDAC	Reference
αVβ6 integrin	Lung cancer, Colorectal cancer, Breast cancer, Cervical cancer, Pancreatic cancer	An integrin member, activates transforming growth factor-β1 (TGF-β1)	Upregulated in a disease context including cancer and fibrosis; up to 100% in well-differentiated tumors, with expression strictly maintained in distant metastases.	High (~80%-100%): Strong expression in the vast majority of PDAC cases.	(131–136)
5T4	AML; Pancreatic cancer, Bladder cancer, Breast cancer, Colorectal cancer, Cervical cancer, Pancreatic cancer, Prostate cancer, Renal cancer, Mesothelioma	Trophoblast glycoprotein, cell surface oncofetal glycoprotein	Expression is associated with poor survival in patients and cancer stem cell niches; closely correlated with advanced clinical stage and metastasis.	High (~50%-80%): Robustly overexpressed across the majority of PDAC tissues.	(137–140)
CEACAM6	Pancreatic cancer, Gastric cancer, Colorectal cancer	A CEA family member, cell surface oncofetal glycoprotein	Affects fibrotic reaction, anoikis resistance, and tumor proliferation. Higher expression levels are associated with a poor prognosis.	High (~90%): Overexpressed in >90% of invasive adenocarcinomas and early lesions; 73-82% high-intensity positivity in clinical pre-screening.	(141–144)
CEACAM7	Pancreatic cancer	A CEA family member, cell surface oncofetal glycoprotein	Highly enriched specific expression within the cancer stem cell compartment and higher expression level correlates with a poor prognosis.	Moderate (~40%-50%): High expression in PDAC. Significantly upregulated in early-stage (Grade 1-2) tumors.	(145, 146)
GPC1	Bladder cancer, Colorectal cancer, Esophagus cancer, Glioblastoma, Ovarian cancer, Pancreatic cancer, Prostate cancer	A glypican family member, cell surface oncofetal glycoprotein	Higher expression associated with perineural invasion and poor prognosis	Moderate (~59.7%): Present in ~60% of primary tumors, 63.6% of early-stage lesions, and dramatically upregulated to near 100% in hepatic and regional metastases.	(147–151)
Eva1	Breast cancer, Hepatic carcinoma, Ovarian cancer, Pancreatic cancer, Respiratory tract cancers, Urinary bladder cancer	A member of the immunoglobulin superfamily expressed on the membrane of developing thymus epithelial cells	Highly expressed on various tumor cell lines and primary tumor cells, also expressed on monocytes, esophagus and salivary glands.	Prognostic biomarker. Statistically differentiates PDAC from adjacent normal tissue with an exceptionally high accuracy (AUC of 0.919).	(152–154)
KRAS G12V	Colorectal cancer, Lung cancer, Pancreatic cancer	An endogenous GTPase	KRAS-G12V mutant neoantigen, broadly targeted by TCR-T or vaccine	Second most common (30.8%) KRAS mutations in PDAC cases.	(155–157)

PDAC, metastatic pancreatic ductal adenocarcinoma; AML, acute myeloid leukemia; AUC, area under the curve.

### 5.1 αVβ6 integrin

The αVβ6 integrin is highly expressed in approximately 90% of PDACs, where it activates TGF-β1, promoting tumor invasion and progression (131). Its low expression in healthy adult epithelium suggests a favorable therapeutic window, and the αVβ6 antibody–drug conjugate (ADC) has advanced Phase 3 clinical trials for Non-Small Cell Lung Cancer (NCT06012435) (132). Preclinical studies show that αVβ6 CAR utilize foot and mouth disease virus-derived A20 peptide for selective αVβ6 targeting, and the CAR-T cells engineered to co-express the chemokine receptor CXCR2 for enhanced homing to tumors or IL-4 receptor ectodomain for T cell proliferation, these two strategies both showed superior anti-tumor activity in pancreatic xenograft models (133, 134).

### 5.2 5T4

The oncofetal glycoprotein 5T4 is highly expressed in PDAC tumors and is associated with poor survival. It is also expressed on cancer stem cells, making it a relevant target for preventing chemoresistance and recurrence (137). While anti-5T4 CAR-T cells were able to kill Acute Myeloid Leukemia (AML) efficiently and specifically killed AML without impacting HSCs (138), they might need further enhancement for PDAC as they showed limited killing in the patient-derived tumor slice model (139).

### 5.3 CEACAM6 and CEACAM7

As members of the CEA family, both CEACAM6 and CEACAM7 are also overexpressed in PDAC and correlated with poor prognosis (141, 142). CEACAM6 is a potential therapeutic target for PDAC which affects fibrotic reaction and tumor proliferation (143). CEACAM7 has low expression in normal tissues and strong surface expression on a subset of primary human PDAC tumors (146). Both CEACAM6 and CEACAM7 CAR-T cells have been developed and their anti-tumor activities have been verified *in vitro* and *in vivo* (145, 146). Targeting these proteins offers a pathway to engage a large proportion of pancreatic tumors.

### 5.4 Glypican-1

GPC1 is a oncofetal glycoprotein of glypicans family. While it is overexpressed on the tumor cell surface of PDAC, GPC1+ cancer exosomes was identified for early pancreatic cancer detection (147). Its elevated expression is associated with perineural invasion and poor prognosis (148, 149). GPC1 CAR-T showed strong antitumor effect in mouse tumor models which can be synergized by PD-1 blockage (150). As GPC1 is a membrane distal epitope, a structure optimized CAR with IgG4 hinge and CD28 transmembrane domain could further improve T cell signaling and tumor killing for PDAC (151).

### 5.5 Epithelial V-like antigen 1

Eva1 is a member of the immunoglobulin superfamily found on various tumor cells, including PDAC. Eva1 can promote tumor progression and metastasis in Hepatocellular Carcinoma (HCC) (152). Its potential as a CAR-T target is complicated by its expression on some normal tissues, including monocytes, esophagus, salivary glands and small intestine. This expression profile raises potential concerns for OTOT. Any clinical development of Eva1-targeted therapies will require sophisticated CAR designs or safety switches to mitigate risk to healthy tissues (153).

### 5.6 KRAS G12V

Nearly all PDAC tumors (~90%) are initiated by an activating mutation in the KRAS oncogene (155). Unlike the surface antigens targeted by CAR-T cells, mutant KRAS creates an intracellular neoantigen that can be presented on the cell surface by MHC molecules. This makes it a prime target for cancer vaccine and TCR-T, which are designed to recognize such intracellular peptides (158, 159). A recent study developed the CAR-T which targets G12V presented by HLA A*11:01, and its antitumor efficacy and safety was improved by TCR deletion and inducible IL-12 expression. This study indicated that CAR-T targets could also break the surface protein limitation to intracellular tumor neoantigens (156).

## 6 Dual-targeting CAR-T strategies for PDAC

**Figure 3:**
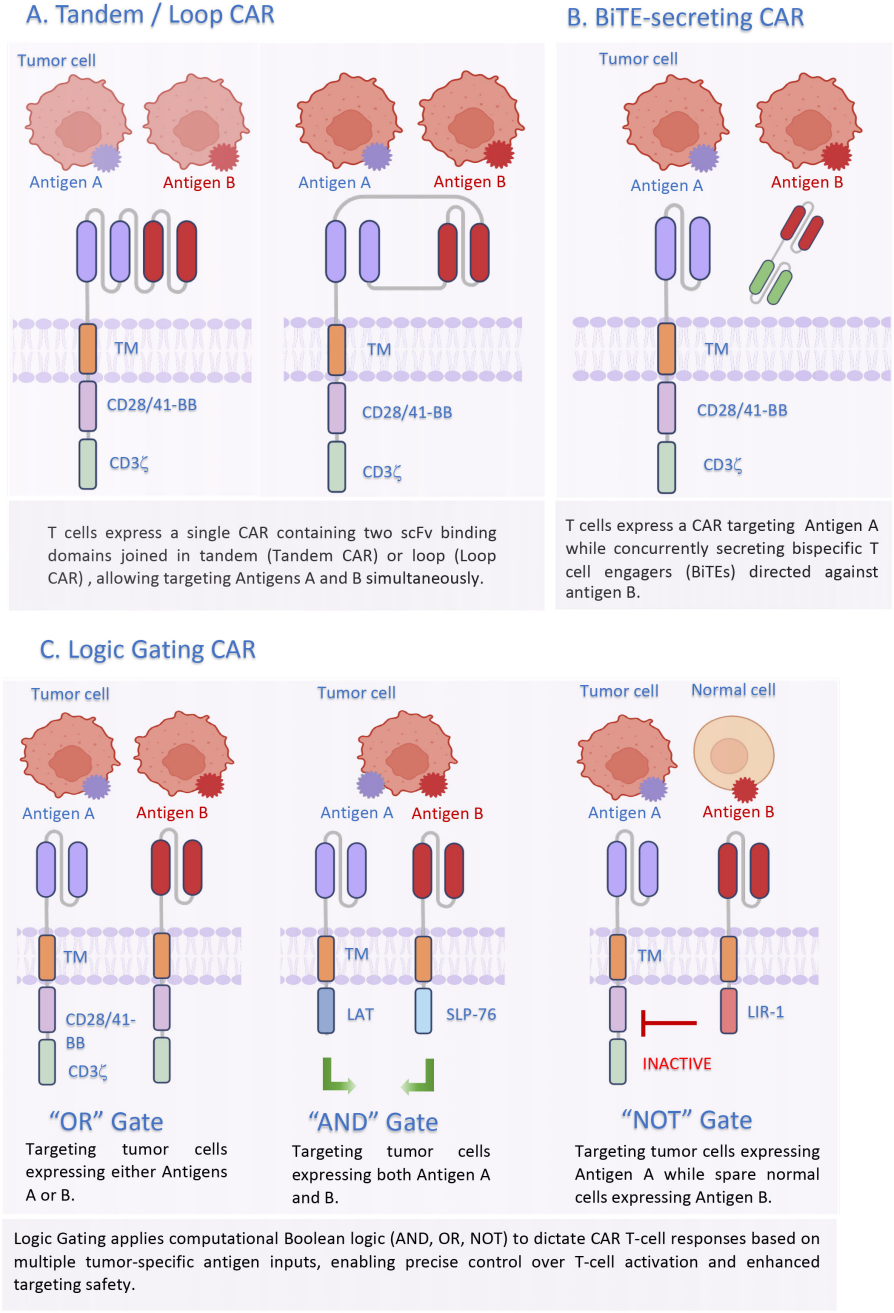
Schematic illustration of dual-targeting CAR-T cell engineering strategies. **(A)** Tandem/Loop CAR: A bispecific single receptor with two antigen-binding domains arranged in tandem or a loop, which can be activated by either antigen. **(B)** BiTE-secreting CAR: CAR T-cells secrete soluble bispecific T-cell engagers (BiTEs) which redirect both the CAR T-cell and bystander T-cells to a second tumor antigen. **(C)** Logic Gating CAR: Simultaneous surface expression of two independent CARs via a single bicistronic vector or by co-transduction with two separate vectors. The Boolean logic circuits such as “OR” gate (full activation by either tumor antigen), “AND” gate (full activation requiring dual tumor antigen recognition) or “NOT” gate (healthy tissue inhibitory safety switch) provides precise control over CAR-T cell activity.

PDAC is a highly heterogeneous disease, and this heterogeneity makes targeting a single antigen insufficient to achieve optimal therapeutic efficacy. Therefore, dual-targeting CARs are engineered to overcome the tumor antigen heterogeneity and escape, enhance CAR-T function, or improve safety profile by preventing OTOT (160). A range of innovative strategies such as Tandem or Loop bispecific CAR, bispecific T-cell engager (BiTE)-secreting CAR, Bicistronic or co-transduction dual CAR with Logic-gating CAR signaling are designed and under investigation from pre-clinical to clinical stages (**Figure 3**). We have summarized the promising dual-targeting CAR approaches for PDAC treatment in **Table 3**, classifying them by designs and their aims to overcome different challenges in solid tumor therapy.

**Table 3:** Potential dual-targeting CAR-T strategies for PDAC.

Targets	Highest phase	Notes/rationale	Reference
CLDN18.2 & NKG2DL	Phase I	Tandem CAR targeting two TAAs, CLDN18.2 and NKG2DL, aiming to overcome antigen heterogeneity and loss.	NCT06134960, NCT05583201 (161, 162),
CLDN18.2 & PD-L1	Phase I	Targeting TAA CLDN18.2 and an immune checkpoint (PD-L1) to address antigen heterogeneity and overcome immune evasion.	NCT06084286
CLDN18.2 & MSLN	Phase I	Two separate CARs targeting CLDN18.2 and MSLN, aiming to overcome antigen heterogeneity and loss.	NCT07066995
GCC & CD19	Phase I	CD19 CAR is co-transduced as a “helper” to boost the expansion and persistence of GCC CAR-T cell	ChiCTR2000040645 (76),
Axl & CD19	Phase I	CD19 CAR is co-transduced as a “helper” to boost the expansion and persistence of Axl CAR-T cell	ChiCTR2400087812
MUC1 & CD19	Phase I	CD19 CAR is co-transduced as a “helper” to boost the expansion and persistence of MUC1 CAR-T cells	ChiCTR1900022608, ChiCTR1900022609
CEA & CD30	Phase I	Targets CEA on tumor cells and CD30 on T cells to “de-repress” CAR-T cells, enhancing their activation and cytotoxic function.	ChiCTR2100053884 (163),
Axl & CD73	Phase I	Targets CD73 and Axl for dual tumor killing and immune modulation.	NCT06939270 (164),
MSLN & HLA-A*02	Phase I	NOT-gated CAR only targeting MSLN positive tumors while sparing HLA-A*02 positive normal tissues to mitigate OTOT.	NCT06682793 (165, 166),
EGFR & HLA-A*02	Phase I	NOT-gated CAR only targeting EGFR positive tumors while sparing HLA-A*02 positive normal tissues to mitigate OTOT.	NCT06051695 (84, 85),
CLDN18.2 & FAP	Preclinical	Targets CLDN18.2-expressing tumor cells and FAP-expressing CAFs to disrupt stromal barriers and immunosuppression.	(167, 168)
GPC3 & FAP	Preclinical	Targets GPC3-expressing tumor cells and FAP-expressing CAFs to disrupt stromal barriers and immunosuppression.	(169)
MSLN & FAP	Preclinical	MSLN-targeted CAR-T cells engineered to secrete FAP/CD3 BiTEs against FAP-expressing CAFs.	(170)
MSLN & NKG2DL	Preclinical	MSLN-targeted CAR-T cells engineered to secrete NKG2D-BiTEs against NKG2DL + tumor cells	(171)
MSLN & MUC16	Preclinical	Tandem CAR targeting two TAAs, MSLN and MUC16, aiming to overcome antigen heterogeneity and escape.	(172, 173)
CEACAM5 & CEACAM6	Preclinical	New 2A3 single-domain antibody-based CAR targeting both CEACAM5 and CEACAM6, aiming to overcome antigen heterogeneity and escape.	(145)
EpCAM & ICAM-1	Preclinical	Bispecific CAR T cells targeting both EpCAM and ICAM-1, aiming to overcome antigen heterogeneity and generate superior antitumor responses.	(174)
CEA & EpCAM	Preclinical	AND-gated CAR targeting both CEA and EpCAM, aiming to overcome antigen heterogeneity and escape, with increased tumor specificity and safety.	(175)
CLDN18.2 & MSLN	Preclinical	AND-gated CAR targeting CLDN18.2 and MSLN mitigate OTOT and promote long-term efficacy.	(176)

### 6.1 Broadening tumor recognition (e.g., CLDN18.2 & NKG2DL, MSLN & MUC16)

The lack of uniformly and highly expressed tumor antigen, known as antigen heterogeneity, in solid tumors is a major cause of immune escape and tumor relapse. Dual-targeting CARs are being designed to recognize usually two distinct TAAs aiming to eradicate all the tumors cells in PDAC. For example, a Phase 1 trial is investigating a tandem CAR targeting both CLDN18.2 and NKG2D ligands (NKG2DLs), stress proteins often upregulated on cancer cells. Similarly, other clinical or preclinical strategies combine targeting of two well-known pancreatic cancer antigens: CLDN18.2/PD-L1, MSLN/MUC16, MSLN/NKG2DL and EpCAM and ICAM-1 et al. (**Table 3**). By targeting two different antigens on one tandem CAR construct (**Figure 3A**), or co-expressing BiTEs (**Figure 3B**), or on separate CAR constructs (**Figure 3C**), these approaches could increase the spectrum of tumor recognition and reduce the likelihood that cancer cells can evade therapy by downregulating a single target (161, 162, 171–173).

### 6.2 Targeting tumor and TME (e.g., MSLN & FAP, CLDN18.2 & PD-L1)

CAFs constitute a major component of the dense desmoplastic TME characteristic of PDAC, contributing significantly to both physical barriers through extracellular matrix remodeling and immunosuppression via the secretion of immunoregulatory factors (129). To disrupt the stroma and tumor cells, dual CAR-T cells are engineered to co-target FAP, the pan-CAF marker with a TAA such as CLDN18.2, MSLN or GPC3 (**Table 3**) (**Figures 3A, B**). The rationale is that the anti-FAP component degrades the stromal matrix, allowing the anti-tumor component better access to the cancer cells (167–170). PD-L1 is elevated on tumor cells and immune suppressive cells in the TME, including TAMs, MDSCs, and Tregs (177, 178). Therefore, PD-L1 CAR-T cell can eradicate both tumor and immunosuppressive cells, and the dual targeting of both PD-L1 and a TAA could further overcome tumor antigen heterogeneity and modulate TME (179),. This combined attacking strategy aims to enhance CAR-T cell infiltration, improve their function within the hostile microenvironment, and ultimately lead to more effective tumor eradication (180). The PDAC TME is also enriched with soluble immunosuppressive factors like TGF-β that drives T-cell exhaustion and promotes fibrosis. TGF-β dominant-negative receptor (DNR) is a truncated receptor binds to TGF-β but lacks the intracellular signaling domain, effectively blocking the downstream suppressive signal (181, 182). A recent trial of TGF-β DNR-armored CAR-T cells (C-CAR031) reported manageable safety and encouraging tumor regression in patients with advanced solid tumors (105), suggesting that dual CAR-T cells can be armored with a TGF-β DNR to further enhance their resistance against the immunosuppressive TME.

### 6.3 Using a “Helper” CAR (e.g., GCC & CD19, CEA & CD30)

One of the main reasons for the high efficacy of CAR-T therapy in hematologic malignancies is that the circulating CAR-T cells can easily access the tumor antigen in the bloodstream and got expanded quickly, while CAR-T cells targeting solid tumors needs to infiltrate into tumor lesions in the first step (183). The “helper” or “booster” CAR strategy addresses this by co-transducing the CD19 CAR with a CAR targeting a solid tumor antigen (like GCC, AXL or MUC1) (**Figure 3C**). The anti-CD19 CAR, by targeting the patient’s normal B-cell population, undergo the robust proliferation and persistence after infusion, providing quick expansion of CAR-T populations. This expansion provides a systemic boost of cytokines and growth factors that support the proliferation and survival of the solid tumor-targeting CAR-T cells (76). Besides the B cell surface protein CD19, simultaneous targeting the CD30 on T cell surface and a TAA is reported to be able to improve T cell activation and anti-tumor response of the CAR-T cells (163).

### 6.4 Logic gating CAR (e.g., CEA & EpCAM, CLDN18.2 & MSLN)

“Logic Gating” CAR is a concept utilizes Boolean operators (AND, OR, NOT) to engineer T cells which addresses not only the challenge of antigen heterogeneity but also OTOT (**Figure 3C**). The “OR” gated CAR-T involves the design of Tandem/Loop CAR and Bicistronic/Co-transduction CAR, which can trigger cytotoxicity by any one of the antigens. In contrast, the “AND” gate requires two tumor antigen for full T cell activation, while the “NOT” logic-gate means the tumor antigen CAR is inhibited by another antigen typically presented on normal cells (184). The logic-gate strategy is particularly critical for PDAC, given that typical targets CLDN18.2 and MSLN are found on gastric mucosa or fibrotic lung, respectively (56, 73). These risks were recently underscored by two studies. Talar Tokatlian et al. designed a “NOT” logic-gate CAR-T engineered with a novel MSLN activating CAR and an HLA-A*02 inhibitory CAR, and this design will spare the healthy tissue that express HLA-A*02 (165). In their Phase 1/2 clinical trial, dose escalation started from 1×10^8^ to 14×10^8^ cells. The ability to administer such high doses, comparable to those used in TIL or TCR-T therapies, is encouraging and will help determine whether this logic-gating strategy can significantly expand the therapeutic window of CAR-T cells (166). Birocchi et al. demonstrated that “AND” gate strategies decouple T-cell activation, requiring the simultaneous binding of both CLDN18.2 and MSLN to trigger cytotoxicity, therefore improve safety and the therapeutic window (176). Their study employed the “AND”-gate technology known as LINK CAR, which pairs the signaling domains of LAT and SLP-76 for full activation (185). Another modular system is called “Dual-RevCAR”, and it achieves complete T cell effector function through triggering CD3 and CD28 signals only upon cross-linking by bispecific target modules recognizing both CEA and EpCAM (175). Applying these combinatorial logic gate technologies to PDAC could enable the safe targeting of potent antigens like CLDN18.2, restricting lytic activity to dual-positive tumor cells and sparing healthy tissues that express only one antigen.

## 7 Conclusion and Future Perspectives

Despite a history of frustrating outcomes for immunotherapy in PDAC, largely due to its classification as an immunologically “cold” tumor, there are reasons for cautious optimism (186). A critical insight has come from the observation that a subset of PDAC patients with a high mutational burden (HMB) can achieve better responses to ICIs (22). A higher mutational load corresponds to a greater number of tumor neoantigens, which can be recognized by the immune system. The success of PD-1/PD-L1 blockade in this specific subgroup indicates that endogenous T-cells possess the intrinsic ability to infiltrate the dense PDAC stroma, recognize tumor antigens, and execute an effective anti-tumor response once immunosuppressive signals are lifted. This finding provides a powerful rationale for adoptive cell therapy (187). If the fundamental principle of T-cell-mediated killing is viable in PDAC, then a strategy like CAR T-cell therapy, which engineers T-cells to potently recognize a specific surface antigen, remains a logical and promising approach to apply this principle to a broader patient population.

The efficacy of CAR-T cell therapy in solid tumors is fundamentally dependent on the density of surface antigen expression. This relationship has been clinically validated in trials such as those for CLDN18.2-targeted CAR-T, where robust responses (ORR ~38.8%) were observed in patients selected for high antigen expression (e.g., ≥40% tumor cells with ≥2+ intensity), and in the GCC19 CAR-T trial for metastatic colorectal cancer, which achieved a 40% objective response rate in patients with confirmed GCC expression (62, 76). Thus, the antigen expression threshold needs to be prioritized for patient selection protocols; however, relying on archival primary tissue for screening often misrepresents the recurrent tumor antigen landscape due to therapy pressure and metastasis. A proportion of patients with initially positive tumors converted to a negative status (known as discordance rate), such as CLDN18.2 in pancreatic cancer (16.7%) (188), TROP2 in brain tumor (21.4%) (189), and HER2 in breast cancer (21.4%) (190). To mitigate the risks of antigen escape, in addition to developing the dual CAR-T strategies, immunohistochemical verification on fresh biopsies of the recurrent lesion is highly recommended for enrollment.

The success of CD19-targeted CAR T-cell therapy in hematological malignancies has set a high benchmark, largely due to the uniform and high expression of CD19 on target cells and the clinically manageable nature of on-target B-cell aplasia. However, translating this success to solid tumors, such as PDAC, is fraught with challenges, chief among them being the lack of true TSAs. The field has instead focused on TAAs, which often have low-level expression on healthy tissues. The selection of a suitable TAA hinges on a delicate balance between maximizing anti-tumor efficacy and minimizing toxicity. Though targets like CLDN18.2, expressed on healthy gastric mucosa, represent a viable strategy where OTOT is considered tolerable, the scFv sequence and signaling domain needs to be carefully evaluated (56). Some targets, such as FAP and MSLN, which are conditionally expressed in fibrotic or wound-healing tissues could result in severe adverse effects (73).

A critical limitation for solid tumor CAR-T therapy is the OTOT, which restricts the administrable cell dose, thereby limiting therapeutic potency. To overcome this, the next generation of CAR-T cells must be more sophisticated. The development of novel targets, combined with multi-targeting strategies (e.g., dual-antigen recognition) and logic-gated CARs (e.g., AND, OR, NOT gates), is paramount. These approaches aim to broaden the spectrum of tumor cell recognition while implementing safety switches to spare healthy tissues. By enhancing tumor specificity and mitigating OTOT, it may become feasible to administer higher, more effective cell doses, potentially approaching the doses used in TCR-T or TIL therapies. It is for this reason that a comprehensive summary of emerging targets and multi-targeting strategies is a central focus of this review.

Another pivotal strategy to enhance CAR-T efficacy is the rational combination with targeted therapies. While CAR-T cells directly target surface antigens, the integration of small molecule inhibitors can synergistically sensitize tumor cells and remodel the immunosuppressive TME (191). For instance, inhibitors targeting DDR, such as PARP inhibitors, have been shown to induce cytosolic DNA accumulation in tumor cells. This event activates the cGAS-STING pathway, triggering the release of Type I interferons and chemokines (e.g., CXCL10) that actively recruit CAR-T cells into the solid tumor bed (191). However, systemic DDR inhibition can also induce DNA damage in T cells, impairing their proliferation and persistence (192). Recent preclinical studies offer robust solutions to this challenge. Optimizing treatment schedules, such as the sequential administration of PARP and WEE1 inhibitors, has been shown to maintain tumor-intrinsic immune activation while sparing T cells from severe DNA damage (193). Alternatively, engineering CAR-T cells to be intrinsically resistant to these inhibitors (for example, by editing the PARP1 gene) preserves their viability and cytotoxic function during combination therapy (192). These strategies suggest that the future of solid tumor treatment lies not just in combining therapies, but in harmonizing them to maximize the therapeutic window.

Ultimately, to unlock the full potential of CAR T-cell therapy for solid tumors, the field must evolve from a T cell-centric to a disease-centric design philosophy. While engineering T cells to overcome physical barriers and the hostile TME remains vital, it is not the complete picture. A more holistic approach should integrate two pillars. First, stringent patient selection is essential; clinical trials should increasingly focus on enrolling patients with high, verified expression of the TAA in the targeted lesions. Second, the future of CAR-T therapy lies in rational combination strategies. Synergistic regimens combining CAR-T cells with conventional treatments like chemotherapy and radiation, which can debulk tumors and release tumor antigens, or with other emerging therapies, such as ADC, BsAb, ICI and oncolytic virus, hold immense promise for enhancing T-cell persistence and function within the TME (4, 180). The NDA approval of the first CAR T-cell product for a solid tumor will undoubtedly be a historic turning point. Such an approval will not represent an endpoint, but rather a critical starting point, catalyzing the clinical investigation of these combinatorial approaches and accelerating the entire research field toward providing meaningful therapeutic options for patients with intractable diseases like PDAC.
